# The underlying neuropsychological and neural correlates of the impaired Chinese reading skills in children with attention deficit hyperactivity disorder

**DOI:** 10.1007/s00787-024-02422-w

**Published:** 2024-04-25

**Authors:** Zhao-Min Wu, Peng Wang, Yun-Yu Zhong, Yun Liu, Xue-Chun Liu, Jiu-Ju Wang, Xiao-Lan Cao, Lu Liu, Li Sun, Li Yang, Yu-Feng Zang, Ying Qian, Qing-Jiu Cao, Yu-Feng Wang, Bin-Rang Yang

**Affiliations:** 1 Shenzhen Childrens Hospital, Shenzhen, China; 2 Shenzhen Pediatrics Institute of Shantou University Medical College, Shenzhen, China; 3 Fuwai Hospital, Beijing, China; 4 Peking University Sixth Hospital/Institute of Mental Health, National Clinical Research Center for Mental Disorders (Peking University Sixth Hospital), Beijing, China; 5 Affiliated Hospital of Hangzhou Normal University, Hangzhou, China

**Keywords:** ADHD, Reading, Executive function, Brain functional connectivity

## Abstract

Impaired basic academic skills (e.g., word recognition) are common in children with Attention Deficit Hyperactivity Disorder (ADHD). The underlying neuropsychological and neural correlates of impaired Chinese reading skills in children with ADHD have not been substantially explored. Three hundred and two children with ADHD (all medication-naïve) and 105 healthy controls underwent the Chinese language skill assessment, and 175 also underwent fMRI scans (84 ADHD and 91 controls). Between-group and mediation analyses were applied to explore the interrelationships of the diagnosis of ADHD, cognitive dysfunction, and impaired reading skills. Five ADHD-related brain functional networks, including the default mode network (DMN) and the dorsal attention network (DAN), were built using predefined regions of interest. Voxel-based group-wise comparisons were performed. The ADHD group performed worse than the control group in word-level reading ability tests, with lower scores in Chinese character recognition (CR) and word chains (WS) (all *P* < 0.05). With full-scale IQ and sustained attention in the mediation model, the direct effect of ADHD status on the CR score became insignificant (*P* = 0.066). The underlying neural correlates for the orthographic knowledge (OT) and CR differed between the ADHD and the control group. The ADHD group tended to recruit more DMN regions to maintain their reading performance, while the control group seemed to utilize more DAN regions. Children with ADHD generally presented impaired word-level reading skills, which might be caused by impaired sustained attention and lower IQ. According to the brain functional results, we infer that ADHD children might utilize a different strategy to maintain their orthographic knowledge and character recognition performance.

**Supplementary Information:**

The online version contains supplementary material available at 10.1007/s00787-024-02422-w.

## Introduction

Attention Deficit Hyperactivity Disorder (ADHD) is a common childhood-onset psychiatric disorder characterized by developmentally inappropriate inattention and/or hyperactivity/impulsivity. Academic failure is common in children with ADHD, which causes excellent parental stress. A previous study demonstrated impaired word length and word frequency in subjects with ADHD [1]. In another study, students with ADHD were found to have more significant difficulties in high-order writing performance compared to their peers [2]. In a community sample, ADHD subjects displayed impaired basic academic skills (e.g., word recognition) and more advanced academic functions (e.g., reading comprehension) [3]. Clinical studies also identified a high comorbidity rate between ADHD and developmental dyslexia (DD, which was estimated to be around 25–40% [4].

In the past decades, several studies have tried to figure out why ADHD subjects tend to have more reading problems. Shared neurocognitive deficits and altered brain function were investigated. Deficits in working memory, which play an essential theoretical role in converting orthographic symbols to phonological sounds, were partly related to impaired reading abilities in subjects with ADHD [5, 6]. Chinese-speaking children with comorbid DD and ADHD also demonstrated more significant deficits in auditory working memory and rapid naming than the ADHD-only or DD-only group [7]. Nevertheless, the mediating effect of working memory on ADHD-related reading difficulty was not validated [8]. In addition, working memory training failed to improve the reading ability of children with ADHD [9]. Instead, sustained attention was shown to be influencing the reading performance in subjects with ADHD [8, 10–12]. A twin study demonstrated that 11% of the shared genetic risk between inattention and RD was captured by short-term memory, and the estimates for working memory (WM) and reaction time variability (RTV, an index of sustained attention) were 21% and 28%, respectively [13]. Inhibition, as well as other executive function components, were also found to be related to the reading ability in subjects with ADHD [14]. A more comprehensive study has demonstrated that multiple executive function deficits were related to reading dysfunction in subjects with ADHD [15, 16]. It is worth mentioning that the Chinese language is different from alphabetic languages. Studies assessing the underlying cognitive profile of the deviated reading ability in children with ADHD, especially in the Chinese readers, are still lacking.

In terms of neural correlates, there have been some studies trying to utilize brain imaging techniques to investigate the brain functional characteristics in subjects with ADHD, DD, or ADHD and DD. Aberrant activity in the frontal cortex [17] and striatum [18], as well as altered functional connectivity in the default mode network, were identified in participants with ADHD, compared to healthy controls [19]. The left middle frontal gyrus was commonly shown to play an essential role in reading ability. In Chinese readers, the right frontal gyrus was found to be as important as the left hemispheric regions [20]. Previous studies have generated inconsistent results in identifying common neural correlates in ADHD and DD. One meta-analytic study failed to identify overlapping structural abnormalities in subjects with ADHD and DD [21]. One previous study demonstrated that those patients with comorbid ADHD and DD showed additive brain functional and structural alterations in the ADHD-only and DD-only group, compared to controls [22]. Another study found out that under-activation in the left-hemisphere reading network were present in both DD and ADHD + DD children [23], which indicated that the ADHD status might not influence reading ability-related brain alterations. This was consistent with results from another study [24]. All these results, taken together, indicated that ADHD and DD might have no overlapping functional correlates and the comorbidity were attributed to additive brain functional alterations. Nevertheless, in some other studies, attention function and reading ability were intertwined. One study showed that functional connectivity within the sustained attention functional network predicted reading ability in a group of healthy individuals [25]. The volume of the left middle frontal gyrus was larger in subjects with DD and ADHD, compared to those with ADHD-only or DD-only, which was not related to basic reading ability but to attention control [26].

In summary, the underlying neuropsychological and brain-functional correlates of the impaired reading skills in ADHD remain unclear. Especially, Chinese is a logographic language, which differs from alphabetic languages, e.g., English. Findings in the English readers might be different from those of the Chinese population. For instance, in previous MRI studies, reading-related brain function networks were shown to be left-lateralized in English readers, while the right hemisphere also plays an essential role in Chinese readers [20]. The current study, therefore, aimed to systematically investigate the Chinese reading skills in children with ADHD and the corresponding neuropsychological and brain-functional characteristics in a group of children with ADHD and typically developing controls.

## Methods

### Participants

Three hundred and two children with ADHD-only (e.g., without comorbidity) were recruited at Shenzhen Children’s Hospital. Another 105 healthy controls from local elementary schools participated in the current study. The inclusion and exclusion criteria can be found in the supplementary material. All psychiatric diagnoses were confirmed or excluded by a clinical interview and a structured interview based on the Schedule for Affective Disorders and Schizophrenia for School-Age Children -Present and Lifetime version (K-SADS-PL) [27] with well-trained specialists according to the Diagnostic and Statistical Manual of Mental Disorders Fourth Edition (DSM-IV). Note that any kind of learning disorders was not specifically excluded in either the ADHD or the control group since there is currently no valid and standardized diagnostic tool for learning disorders in China.

This work was approved by the Ethics Committee of Shenzhen Children’s Hospital (identification code: 202,204,202). Written informed consent was obtained from parents and children.

### Reading skills and cognitive assessment

For both the ADHD and the control group, the Chinese reading skill tests, adopted from the prior work of Shu et al. (2006) [28] and Liu et al. (2017) [29], were carried out by well-trained psychologists. This test provided detailed information about Chinese reading skills. The outcome measures are ① Chinese character recognition (CR): the subject is asked to read out the Chinese characters they know. ② orthographic knowledge (OT): This measures visual-orthographic processing. The subject was asked to identify non-characters. The number of correct answers by the subject was recorded and used as the OT measure in the current study. A previous study has shown that orthographic knowledge strongly predicted reading proficiency levels. ③ Word Chains (WS): it required the subject to put a slash to clusters of words presented as a continuous line of print without inter-word spaces, separating into three meaningful words. The number of correct answers by the subject was recorded and used as the WS measure in the current study. The Wechsler Intelligence Scale for Chinese Children-IV (WISC-IV) was applied to rule out intelligence disability. Besides, the Cambridge Neuropsychological Test Automated Battery (CANTAB), including Spatial Working Memory (SWM), Reaction Time (RTI), Stop Signal Task (SST), and Rapid Visual Information Processing (RVP), was conducted to assess the cognitive function in each subject. More details about these CANTAB tests can be found in the supplementary material.

### Statistical analysis of the neuropsychological measures

Between-group comparisons were performed on each CANTAB, WISC-IV, and Chinese reading skill measure. Analyses were done using ANCOVA models, with age and sex as covariates. Significant level was set to a nominal two-tailed p-values of < 0.05. Correction for multiple comparisons was not utilized in the first step since we wanted to explore as many potential variables as possible. For those measures that showed norminally significant between-group differences, mediation models were built. In the first step, simple mediation models (using the mediation package in R) with one CANTAB or WISC-IV full-scale IQ as a single mediator, the diagnosis (ADHD or healthy control) as the independent variable, and the Chinese reading skill measures as dependent variables. In the second step, more complicated models (Structural Equation Modeling, using the lavaan package in R) were explored, with full-scale IQ and CANTAB measures as mediators, the diagnosis (ADHD or healthy control) as the independent variable, and the Chinese reading skill measures as dependent variables. Age and sex were also included as covariates in the mediation and structural equation models. Significant level was set to a two-tailed p-values of < 0.05. Bonferroni correction was applied according to the number of measures from the same task tested in the mediation models, and therefore, for the RTI and SST measures, it was set at 0.05/3 = 0.017, and for the RVP measures, it was set to 0.05/2 = 0.025.

### Analytic steps of the rs-fMRI scans

A total of 175 subjects underwent resting-state functional magnetic resonance imaging (rs-fMRI) scans. All rs-fMRI images were processed in the FSL software (https://fsl.fmrib.ox.ac.uk/fsl/fslwiki/FSL) and Python (https://www.python.org/). Details about imaging protocols and preprocessing steps can be found in the supplementary material. Five brain networks, including the default mode network (DMN), the dorsal attention network (DAN), the ventral attention network (VAN), and the executive control network (ECN) were built using predefined regions of interest (ROIs). Details about the ROIs and the figures of each brain network can be found in our previous publication [30]. We have chosen to focus on these five brain networks since they have been previously identified to be involved in the pathophysiology of ADHD, and were related to th severity of core ADHD symptoms [30, 31].

Group-wise comparisons and regression models were performed using the dual-regression and Randomise frameworks in FSL. The between-group voxel-wise analysis was performed with the diagnosis as an independent variable and sex and age as covariates. To explore the Chinese reading skill-related brain functional connectivity (FC) characteristics, with one of the Chinese reading skill measurements as an independent variable and diagnosis as another independent variable. Sex and age were also added as covariates. Note that, in this step, the interaction between any Chinese reading skill measurement and the diagnosis was explored. If the interaction variable turned out to be significant, relationships between the Chinese reading skill measurements and brain FC were explored in the two diagnostic groups separately. If the interaction variable was not statistically significant, the relationships between the Chinese reading skill measurements and brain FC were explored in the whole group. Sensitivity analyses with the head motion parameter (indicated by root mean squared volume to volume displacement were also performed in the abovementioned models. Significant level was set to a two-tailed p-values of < 0.05 (family-wise error (FWE) corrected at the voxel level with Threshold-Free Cluster Enhancement (TFCE)).

Regression models were built to test the relationships between brain FC and neuropsychological measures. For those brain clusters showing group-wise differences (ADHD vs. Controls), the mean FC values were extracted, and regression models were built with Chinese reading skill measures and age and sex as covariates. For those brain clusters showing significant correlations with any Chinese reading skill measures, the mean FC values of these clusters were compared between the ADHD and the control group. Regression models of these mean FC values were also built with neuropsychological measures, such as full-scale IQ and the CANTAB index.

## Results

### The demographic characteristics of the ADHD and the control group

The final sample consists of 302 ADHD subjects and 105 healthy controls. In the sub-sample with fMRI scans, there are 84 ADHD subjects and 91 healthy controls. Compared with the control group, the ADHD group has more male participants (*p* < 0.001), and the ADHD subjects are younger than the control group (*p* < 0.001). More details can be found in Table 1. In the sub-sample with fMRI images, between-group differences in age and sex were similar to those in the full cohort. In addition, there is no significant difference between the ADHD and the control group in the head motion parameter (indicated by root mean squared volume to volume displacement). Details can be found in the supplementary material.

**Table 1:** The demographic characteristics of the ADHD and the control group

	ADHD	HC	t/F/χ^2^ statistics	P
Sample size	302	105	N.A.	N.A.
Male	257(85.10%)	60((57.14%)	35.36	< 0.001
Age	8.29 ± 1.68	9.38 ± 1.32	6.74	< 0.001

Abbreviations: ADHD = attention deficit hyperactivity disorder; HC = healthy control

### The reading skill and neuropsychological characteristics of the ADHD and the control group

The ADHD group showed significantly worse Chinese reading skills, with lower scores in Chinese character reignition (CR) (*p* = 0.0075) and word chains (WS) (*p* = 0.0045). Interestingly, the between-group difference was not significant in terms of orthographic knowledge (OT) (*p* = 0.069). In terms of the WISC-IV index, as expected, the ADHD group has lower full-scale IQ (*p* < 0.001), Verbal Comprehension Index (VCI) (*p* < 0.001), Perceptual Reasoning Index (PRI) (*p* = 0.0018), Working Memory Index (WMI) (*p* < 0.001), and Processing Speed Index (PSI) (*p* < 0.001). Outcomes from CANTAB showed that the ADHD group displayed worse processing speed and sustained attention, with lower RVPA (*p* = 0.041), RVPML (*p* = 0.0054), RTISMDRT (*p* = 0.0015), RTIFMDRT (*p* < 0.001), and RTIFRTSD (*p* = 0.029) scores. Besides, the ADHD subjects also presented worse response inhibition, with higher SSTSSRT (*p* = 0.032), SSTDEG (*p* < 0.001), and SSTDES(*p* < 0.001). More details can be found in Table 2. In the sub-sample with fMRI images, worse performance in the Chinese reading skill tests, WISC-IV tests, as well as SST and RTI tests from CANTAB were also observed in the ADHD group compared with the control group. In addition, a lower OT (*p* = 0.0011) score was also present. Details can be found in the supplementary material.

**Table 2:** Reading skill and neuropsychological characteristics of the ADHD and the control group

	measure	ADHD	HC	t/F/χ^2^ statistics	P
Chinese Reading Skill	CR	69.09 ± 39.05	105.67 ± 23.59	7.23	0.0075
	OT	24.48 ± 8.06	30.78 ± 6.14	3.32	0.069
	WS	9.92 ± 7.02	17.33 ± 6.02	8.18	0.0045
WISC-IV	Full-scale IQ	94.26 ± 10.86	104.56 ± 9.28	56.97	< 0.001
	VCI	95.81 ± 11.82	102.37 ± 10.21	25.02	< 0.001
	PRI	101.40 ± 11.74	106.50 ± 10.61	9.91	0.0018
	PSI	93.44 ± 11.74	107.05 ± 11.37	68.92	< 0.001
	WMI	89.39 ± 10.40	97.44 ± 10.10	38.13	< 0.001
RVP	RVPA	0.74 ± 0.09	0.79 ± 0.09	4.22	0.041
	RVPML	639.04 ± 172.30	583.40 ± 108.04	7.86	0.0054
SWM	SWMS	8.68 ± 1.87	8.33 ± 2.11	1.68	0.20
	SWMBE	20.14 ± 7.59	16.49 ± 8.67	2.29	0.13
SST	SSTSSRT	373.77 ± 88.16	337.07 ± 82.42	4.65	0.032
	SSTDEG	32.31 ± 25.42	14.07 ± 16.37	19.70	< 0.001
	SSTDES	50.62 ± 8.12	46.78 ± 8.05	7.79	< 0.001
RTI	RTISMDRT	451.16 ± 112.39	381.11 ± 60.69	10.23	0.0015
	RTIFMDRT	512.42 ± 93.25	428.87 ± 61.29	25.10	< 0.001
	RTISRTSD	136.70 ± 170.39	71.41 ± 38.02	2.62	0.11
	RTIFRTSD	131.57 ± 106.76	84.96 ± 49.63	4.82	0.029

Abbreviations: ADHD = attention deficit hyperactivity disorder; HC = healthy control; CR = Chinese character recognition; OT = orthographic knowledge (OT); WS = Word Chains; VCI = Verbal Comprehension Index; PRI = Perceptual Reasoning Index (PRI); WMI = Working Memory Index; PSI = Processing Speed Index (PSI); RVPA = Rapid Visual Information Processing (RVP), A prime; RVPML = Rapid Visual Information Processing (RVP), mean response latency; SWMS = Spatial Working Memory (SWM), strategy; SWMBE = Spatial Working Memory (SWM), between errors; SSTSSRT = Stop Signal Task (SST), stop signal reaction time; SSTDEG = Stop Signal Task (SST), direction errors: go trials; SSTDES = Stop Signal Task (SST), direction errors: stop trials; RTISMDRT = Reaction Time (RTI), reaction time of the simple mode; RTIFMDRT = Reaction Time (RTI), reaction time of the five-choice mode; RTISRTSD = Reaction Time (RTI), the standard deviation of reaction time of the simple mode; RTIFRTSD = Reaction Time (RTI), the standard deviation of reaction time of the five-choice mode;

### The structure of mediation models

**Fig. 1:**
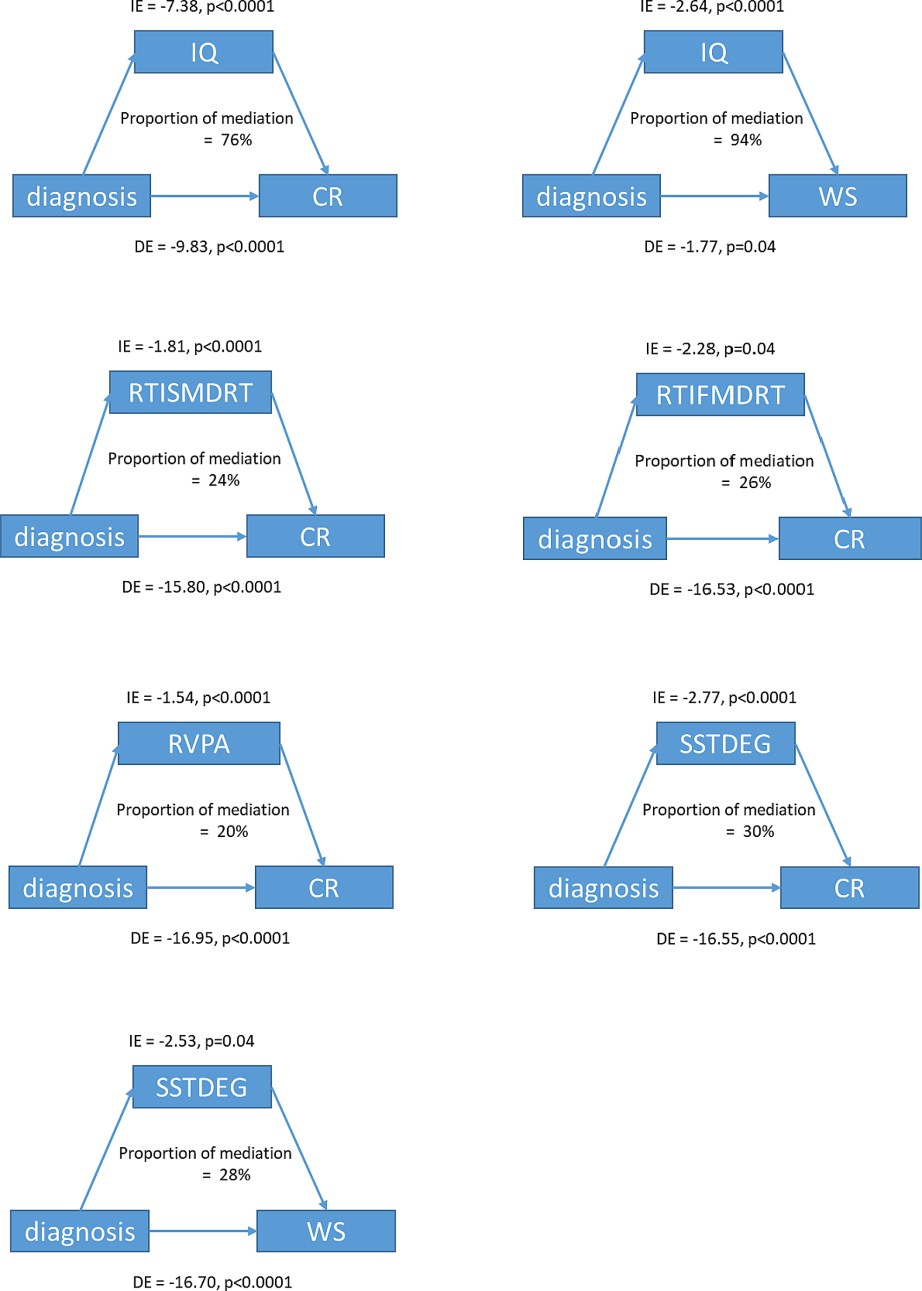
The mediating effects of IQ and other neuropsychological measures on the ADHD status-reading ability relationships. Abbreviations:IE: indirect effects; DE: direct effects; CR: Chinese character recognition; WS: Word Chains; RTISMDRT: Reaction Time (RTI), the reaction time of the simple mode; RTIFMDRT: Reaction Time (RTI), the reaction time of the five-choice mode; RVPA: Rapid Visual Information Processing (RVP), A prime; SSTDES: Stop Signal Task (SST), direction errors: go trials; SSTDEG: Stop Signal Task (SST), direction errors: stop trials

In the first step, with only one mediator in the model, the results showed that the full-scale IQ significantly mediates the relationship between diagnosis and Chinese reading skills, including the Chinese character recognition and word chains (WS) scores (indirect effects *p* < 0.0001). Besides, the neuropsychological index, including RTISMDRT (indirect effects *p* < 0.0001), and RVPA (indirect effects *p* < 0.0001), significantly mediates the relationship between diagnosis and Chinese character recognition score. More details can be found in Fig. 1.

**Fig. 2:**
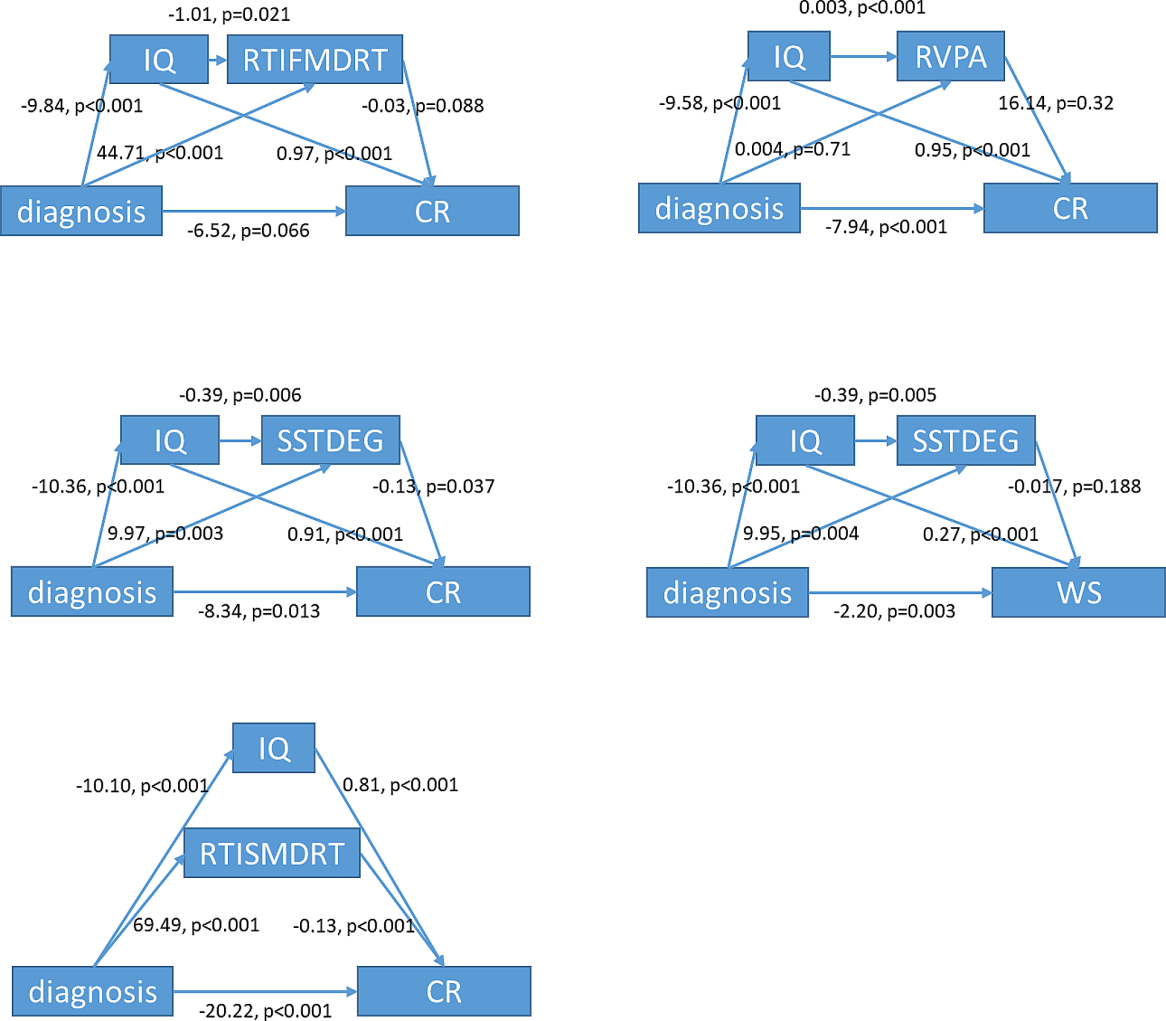
The mediating effects of IQ and other neuropsychological measures on the ADHD status-reading ability relationships, with multiple mediators included in the same model. Abbreviations: CR: Chinese character recognition; WS: Word Chains; RTISMDRT: Reaction Time (RTI), reaction time of the simple mode; RTIFMDRT: Reaction Time (RTI), reaction time of the five-choice mode; RVPA: Rapid Visual Information Processing (RVP), A prime; SSTDES: Stop Signal Task (SST), direction errors: go trials; SSTDEG: Stop Signal Task (SST), direction errors: stop trials

In the second step, with full-scale IQ and neuropsychological index as mediators in the same model. The mediating effects of full-scale IQ and neuropsychological indexes, such as RTIFMDRT, RVPA, SSTDEG, and RTISMDRT, were significant. Note that, when full-scale IQ and RTIFMDRT (a measure of sustained attention) were in the same model, the direct effect of the diagnosis of ADHD on CR became insignificant (direct effects *p* = 0.066). More details can be found in Fig. 2.

### Groupwise differences in brain functional connectivity

Compared with the control group, the ADHD group displayed reduced FC in the left putamen within the DMN and mainly in the right frontal and parietal lobes within the ECN (details in supplementary material sTable two and sFigure 1). These results remained intact in the sensitivity analysis with the head motion parameter as an additional covariate. Details can be found in the supplementary material.

### Reading ability-related functional connectivity characteristics

**Fig. 3:**
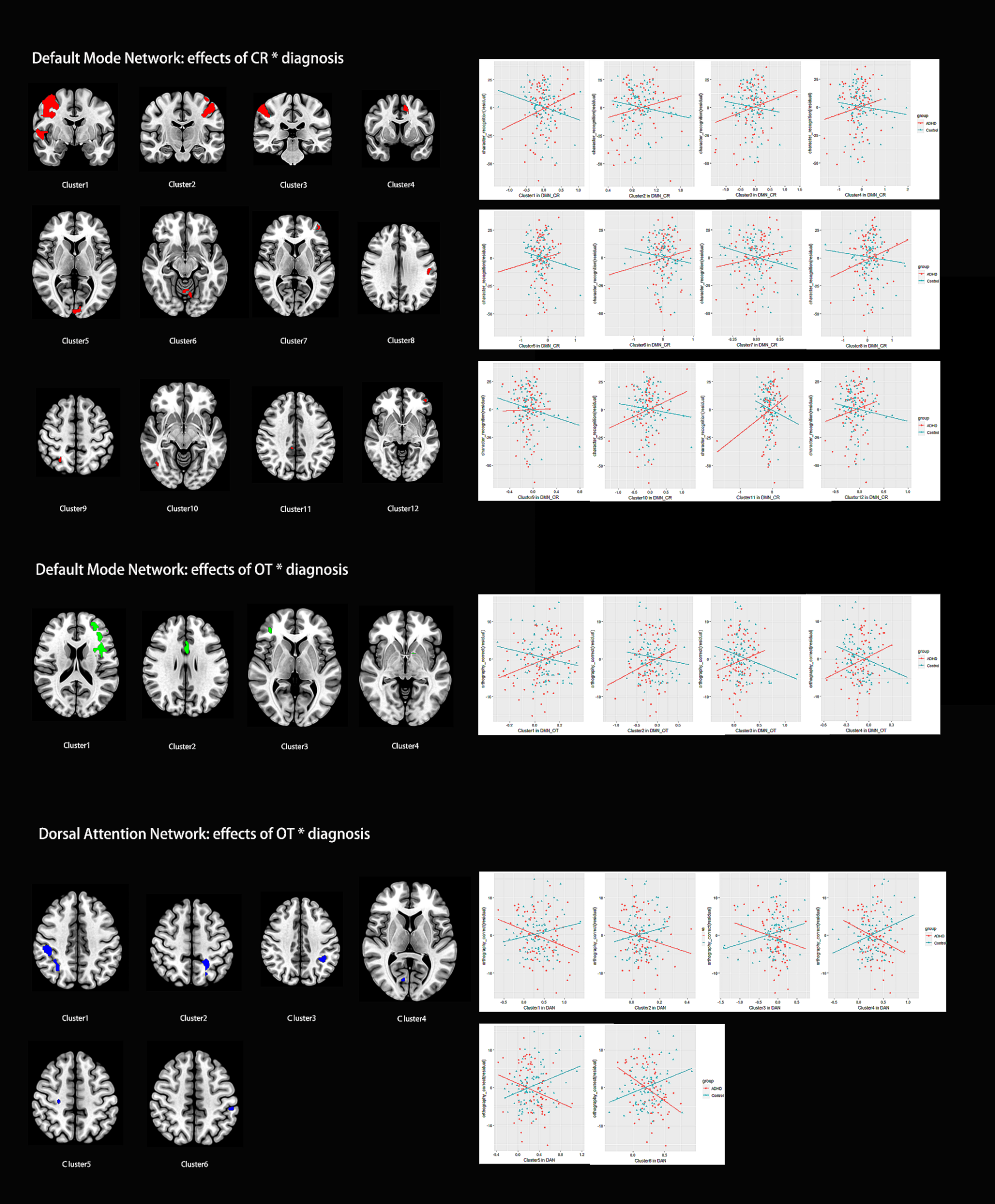
The spatial maps of the clusters showing significant reading ability-brain function correlations displayed on an MNI_T1_2mm_brain. This figure also shows the correlations between the mean functional connectivity within these clusters and the reading ability measures in the ADHD or control group separately. Abbreviations: CR: Chinese character recognition; OT: orthographic knowledge (OT)

The interaction effects of the diagnosis of ADHD and the reading skill measure, including CR and OT, were detected in the DMN, mainly in the frontal lobe and parietal lobe, and the DAN, such as the bilateral postcentral gyrus, bilateral parietal lobule, right lateral occipital cortex, precuneus cortex, and right parietal operculum cortex. More details can be found in Table 3; Fig. 3. Notably, those regions showing interacting effects do not overlap with those showing between-group (ADHD vs. Control) differences. In the control group, the CR and OT scores were negatively correlated with the FC in multiple regions within the DMN. The OT scores were positively correlated with the FC in multiple regions within the DAN. In the ADHD group, the CR and OT scores were positively correlated with the FC in multiple regions within the DMN, and the OT scores were negatively correlated with the FC in multiple regions within the DAN. In the sensitivity analysis with the head motion parameter as an additional covariate, these results remained significant. Details can be found in the supplementary material.

**Table 3:** Clusters showing ADHD diagnosis-reading ability interacting effects

Brain networks	comparisons	Cluster	Voxels	coordinates	regions
DMN	CR_interaction	1	4130	44 4 30	Right frontal pole, right insular cortex, right middle frontal cortex, right inferior frontal gyrus, right precentral gyrus, right postcentral gyrus, right central opercular cortex, right planum polare, right Heschl’s gyrus, right planum temporale;
		2	2739	-32 10 32	Left frontal pole, left superior frontal gyrus, left inferior frontal gyrus, left precentral gyrus, left postcentral gyrus;
		3	1618	58 − 30 54	Right postcentral gyrus, right superior parietal lobule, right supramarginal gyrus, right angular gyrus, right lateral occipital cortex;
		4	173	-10 4 46	Left supplementary motor cortex, left paracingulate gyrus, anterior cingulate gyrus;
		5	164	2–96 8	Right intracalcarine cortex, right cuneal cortex, right occipital pole, right supracalcarine cortex;
		6	88	-6 -56 -24	Left lingual gyrus;
		7	54	-42 54 6	Left frontal pole;
		8	50	-52 -22 28	Left supramarginal gyrus, left postcentral gyrus;
		9	38	26–58 54	Right superior parietal lobule, right lateral occipital cortex;
		10	34	46–66 -10	Right lateral occipital cortex;
		11	21	6–36 36	Posterior cingulate gyrus;
		12	20	-44 48 − 6	Left frontal pole;
DMN	OT_interaction	1	1871	-28 18 6	Left frontal pole, left insular cortex, left middle frontal gyrus, left inferior frontal gyrus, left precentral gyrus, left frontal orbital cortex, left frontal operculum cortex, left central opercular cortex, left planum polare;
		2	365	-2 16 24	Anterior cingulate gyrus, left paracingulate gyrus;
		3	154	42 42 10	Right frontal pole; right inferior frontal gyrus, right middle frontal gyrus;
		4	11	-12 6–8	Left putamen, left pallidum, left accumbens;
DAN	OT_interaction	1	2335	46 − 30 32	Right postcentral gyrus, right superior parietal lobule, right supramarginal gyrus, right lateral occipital cortex, precuneous cortex, right parietal operculum cortex;
		2	347	-22 -52 52	Left postcentral gyrus, left superior parietal lobule, left lateral occipital cortex;
		3	204	-38 -48 38	Left postcentral gyrus, left superior parietal lobule, left supramarginal gyrus, left angular gyrus;
		4	33	10–76 10	Right intracalcarine cortex;
		5	25	24–24 48	Right precentral gyrus;
		6	24	-54 -32 46	Left supramarginal gyrus;

Abbreviations: ADHD = attention deficit hyperactivity disorder; HC = healthy control; CR = Chinese character recognition; OT = orthographic knowledge (OT); DMN = default mode network; DAN = dorsal attention network

### Brain function-cognition relationship

Further analysis showed that the mean FC of those clusters showing interaction effects were not significantly related to IQ and neuropsychological measures, with the exception of the correlation between RVPA and Cluster 3 within DAN (beta = 1.57, *p* = 0.0047) in the control group. The mediation model using the RVPA as a mediator between FC within Cluster 3 of DAN and orthographic knowledge was not significant. The mean FC of those clusters showing between-group differences do not correlate with any of the reading ability measures.

## Discussion

Our results identified impaired reading ability in children with ADHD, characterized by difficulties in word-level reading, e.g., lower scores in CR and WS, with the absence of abnormal orthographic knowledge, which is the fundamental deficit in DD. Cognitive dysfunction in the sustained attention and inhibition domains presented significant mediating effects in the ADHD status-reading difficulty relationship. In terms of brain function, the underlying brain-reading ability correlations were different between the ADHD and the control group.

The current study validated the impaired reading ability in children with ADHD. Nevertheless, we found out that the reading deficit in children with ADHD is restricted to word-level ability. Subjects with ADHD did not present significant deviation from the control group in the orthographic knowledge measure, which is consistent with a previous study from Hong Kong. In that study, the deficiency of orthographic knowledge was more severe in children with DD-only than in those with ADHD and DD [7]. Since the DD group was defined by word-level reading disability, this result might reflect that the diagnosis of ADHD would lead to more word-level reading difficulty without bringing more deficiency in orthographic knowledge. Another study from mainland China also demonstrated similar orthographic legality between children with ADHD and those with comorbid ADHD and DD [32]. In contrast, some studies revealed impaired orthographic knowledge in children with ADHD [8, 33]. The inconsistency in these studies might be partly attributed to the different tasks and measures utilized in each research [34, 35]. On the other hand, as a logographic language, the Chinese language is fundamentally different from English, which might also explain the discrepancy among these results. Note that, in our sub-sample with fMRI images, we also observed a lower OT score in the ADHD group than the control group, which might be attributed to the heterogeneity in ADHD and the fact that we were not able to exclude those comorbid with developmental dyslexia.

In terms of cognitive function, we identified impaired sustained attention and response inhibition in children with ADHD. In mediation models, the full-scale IQ score and the cognitive factors were found to be significant mediating factors between ADHD status and word-level ability. In factor, the proportion of mediating effects of IQ was estimated to be around 94% in the relationship between the ADHD diagnosis and the word-chains (WS) measure. In a complicated model including both IQ scores and advanced sustained attention measure (RTIFMDRT), the direct effect of the ADHD diagnosis on the character recognition score (CR) became statistically insignificant (*p* = 0.066). The fact that IQ partly explained the reading disability in subjects with ADHD was supported by results from neuropsychological and genetic studies [36–38]. A previous study revealed that measures of attention partly accounted for oral language, reading, and writing skills [39] in a group of children with or without specific learning disabilities. Another study also identified attention control as an effective predictor of reading fluency in a mixed sample of children [11]. A longitudinal study has also validated that attention function at five months old is related to reading performance at age 6 [40]. Upon using mediation models, central executive (similar to attention control) and orthographic conversion were revealed to jointly mediate ADHD-related reading comprehension differences entirely [8]. In the Chinese language, as we mentioned above, orthographic knowledge was not impaired in children with ADHD compared to healthy controls. Therefore, in the current study, we took into account the effects of lower IQ and weaker sustained attention in subjects with ADHD and found that explained the entire deficiency in word-level reading disability in the ADHD group. This result indicated that lower IQ and impaired attention function together might be the underlying neuropsychological correlates of the word-level reading difficulties in children with ADHD.

In the current study, we also investigated the brain functional characteristics related to reading ability. The results demonstrated that the underlying brain-reading performance correlation is different between the ADHD and the control group. In terms of orthographic knowledge (OT), a negative correlation between OT and functional connectivity (FC) with default mode network (DMN, mainly the left frontal regions) was present in the control group, while a positive counterpart was identified in dorsal attention network (DAN, mainly the right parietal-occipital regions). A reversed correlation pattern was identified in the ADHD group. Different types of orthographic coding were related to brain activation in different regions [41]. In a group of skilled Chinese readers, activation of the right posterior parietal cortex was found to be associated with orthographic awareness [42], which is in line with what we identified in the current study. The right parietal regions were shown to be involved in decoding relatively easy words [43].

An early fMRI revealed an orthographic processing-related brain network, including the left occipitotemporal region and right middle frontal gyrus [44] in Chinese readers. The left superior parietal lobule and left middle frontal gyrus were also revealed to be essential in orthographic-to-phonological mapping [45, 46]. An EEG study demonstrated that a left-lateralized network was activated in early orthographic processing, while a right-lateralized network was involved later for higher-level processing of orthographic information [47]. Previous MRI studies also demonstrated that the reading strategy of Chinese readers affected their brain functional networks [48, 49]. In line with this, the current study found that the orthographic processing ability was positively correlated with the functional connectivity (FC) within the dorsal attention network, mainly involving the right parietal-occipital cortex, and negatively correlated with the FC within the default mode network, mainly located in the left frontal cortex. This relationship pattern became negative in the ADHD group, which indicates that the ADHD subjects might utilize a different strategy for processing orthographic information.

The character recognition score in the control group is negatively correlated with functional connectivity within the default mode network, mainly in the bilateral frontal cortex. This relationship became positive in the ADHD group. The bilateral frontal cortex was previously identified to be involved in recognizing Chinese characters [50–52]. Spaced and unspaced sentence reading were related to different brain functional networks [53], and the coordination between the visual-word regions and dorsal attention regions plays an important role in grouping characters [54]. Taking into account the fact that the ADHD subjects presented lower CR scores than the control group, we infer that the different word-level reading ability and brain functional network relationships might be attributed to the decompensation mechanism. To finish the reading task, the ADHD group tended to activate more of the DMN regions, and the control group seemed to activate more of the DAN regions. That means the ADHD subjects adopted a different strategy and maintained a close-to-normal level of orthographic knowledge. When they tried to adopt a different way to decode the Chinese characters, they failed. Note that the FC strength of these regions showing different reading ability-brain relationships presented no correlation with sustained attention measures.

### Limitations

The current study has viewed the reading test results as continuous traits rather than diagnostic “cut-offs” since we noticed a discrepancy between a clinical diagnosis of “specific learning disorder” based on DSM-5 [55] and a diagnosis of developmental dyslexia (DD) based on their Chinese reading test results using the cut-off values proposed by the recent expert advice on diagnosis and intervention of Chinese DD in our sample [56]. Future studies using standardized academic tests and validated diagnostic tools to define DD and explore the brain characteristics of ADHD and DD are warranted. Our final model did not include grade as a factor, but this did not indicate that education level was not important. Since repeating a school year is not possible in our current education system, age is, therefore, highly correlated with grade (education level). Adding age and grade in the same model would end with a very high variance inflation factor (VIF), which indicates collinearity. Therefore, only age was added as a surrogate index for education in our analytic models.

## Conclusions

In summary, the current study validated the word-level reading difficulty in children with ADHD. Further analysis using mediation models demonstrated that the attention function was an effective factor in mediating the effects of ADHD status on word-level reading ability. Brain functional MRI revealed that the underlying neural correlates for the orthographic knowledge and character recognition were different between the ADHD and the control group. The ADHD group tended to recruit more DMN regions to maintain reading performance, while the control group seemed to utilize more DAN regions by exploring the underlying neuropsychological and neural correlates of the impaired function.

The underlying neuropsychological and neural correlates of the impaired Chinese reading skills in children with attention deficit hyperactivity disorder.

## Electronic supplementary material

Below is the link to the electronic supplementary material.

Supplementary Material 1

## Data Availability

No datasets were generated or analysed during the current study.
