# Supplementary material for: The underlying neuropsychological and neural correlates of the impaired Chinese reading skills in children with attention deficit hyperactivity disorder
Source: Eur Child Adolesc Psychiatry. 2024 Apr 25;33(11):3979–92. doi: 10.1007/s00787-024-02422-w (PMC11588871; doi:10.1007/s00787-024-02422-w)
Supplement: Supplementary file 1 — Supplementary Material 1 [file 787_2024_2422_MOESM1_ESM.docx]

**Supplementary material**

*Participants*

The inclusion criteria for the ADHD group are as follows:

1. A diagnosis of ADHD;
2. Aged 6-15 years;
3. Normal full-scale intelligence quotient (IQ) measured by the Wechsler Intelligence Scale for Chinese Children-IV (WISC-IV) (≥70);
4. Educated in public or ordinary private school;

The exclusion criteria for the ADHD group include:

1. A history of head injury with loss of consciousness;
2. Any drug or substance misuse;
3. A present or past diagnosis of other psychiatric disorder;
4. History of using prescribed medications for ADHD or other psychiatric disorders;

As for the control group, the inclusion and exclusion criteria are the same as the ADHD group except for the diagnosis of ADHD.

For those who underwent the MRI scans, the following additional criteria should be met:

1. Right-hand dominant;
2. No visible brain abnormalities on the MRI images, e.g., arachnoid cyst;
3. No past or current history of claustrophobia;

*Clinical and Cognitive assessment*

The Cambridge Neuropsychological Test Automated Battery (CANTAB) was conducted to assess the cognitive function of each subject. The CANTAB was a cloud-based platform, and it was administered automatically. The following tests were applied:

① Spatial Working Memory (SWM): the screen presents a number of colored boxes, and the subject is required to find a hidden yellow token by selecting the boxes. A token would not be hidden in the same box twice. This task generates two major measures, the spatial working memory strategy (SWMS) and the spatial working memory between errors (SWMBE). The SWMS reflects the ability of an individual to adopt a certain searching strategy. The SWMBE is the number of times an individual incorrectly revisits a box whose token has been taken.

② Reaction Time (RTI): One (simple mode) or five (five-choice mode) circles appear on the screen. The participant has to hold the button at the bottom of the screen. A yellow dot will appear in one of the circles, and the participant is asked to release the button and select the circle with the yellow dot. This task generates four major measures: the median reaction time in simple mode (RTISMDRT) and the five-choice mode (RTIFMDRT), and the standard deviation (SD) of reaction time in simple mode (RTISRTSD) and the reaction time in five-choice mode (RTIFRTSD).

③ Stop Signal Task (SST): an arrow will appear in the middle of the screen, pointing either to the left or right. The subject must then select either the left or the right button according to the arrow. They will have to withhold making that response when they hear an audio tone (a beep). This task generates three major measures: the stop signal task reaction time (SSTSSRT), the direction errors during go trials (SSTDEG), and the direction errors during stop trials (SSTDES). SSTSSRT is the time where an individual can successfully inhibit half of their responses.

④ Rapid Visual Information Processing (RVP): a digit ranging from 2 to 9 will appear in a pseudo-random order within a white box in the center of the screen. Participants must detect target sequences (e.g., 3-5-7) and respond by pressing the button at the bottom of the screen. This task generates the following major measures: the rapid visual information processing speed A prime (RVPA), which reflects the individual’s sensitivity to the target regardless of response latency, and the mean response latency (RVPML).

More details of CANTAB tests can be found in our previous publication (PMID: 35470726) and on the CANTAB website (<https://cambridgecognition.com/>).

sTable 1: Summary of the CANTAB tasks

| Tasks | Cognitive domains | Measures |
| --- | --- | --- |
| Spatial Working Memory (SWM) | Spatial working memory | SWMS |
|  |  | SWMBE |
| Reaction Time (RTI) | Sustained attention | RTISMDRT |
|  |  | RTIFMDRT |
|  |  | RTISRTSD |
|  |  | RTIFRTSD |
| Stop Signal Task (SST) | Response inhibition | SSTSSRT |
|  |  | SSTDEG |
|  |  | SSTDES |
| Rapid Visual Information Processing (RVP) | Processing speed | RVPA |
|  |  | RVPML |

*Imaging protocols*

A total of 175 subjects underwent resting-state functional magnetic resonance imaging (rs-fMRI) scans. These images were acquired in a 3T Siemens Skyra scanner with a standard 12-channel head coil in Shenzhen Children’s Hospital. Parameters of the rs-fMRI are as follows: Single-shot echo-planar imaging (EPI) sequences were applied: TR=2000ms, TE=30ms, flip angle=90°, thickness/skip=3.5/0.7mm, matrix=64×64, field of view (FOV)=200×200mm, 33 axial slices, 240 volumes, and 3mm×3mm in-plane resolution. High-resolution 3D T1-weighted images were also collected, using a magnetization-prepared rapid gradient echo (MPRAGE) sequence: 176 sagittal slices, TR/TE = 2530/2.25 ms, flip angle = 7°, FOV = 256 × 256 mm, 1.33 mm thickness with no gap, 1 × 1 mm in-panel resolution.

*Preprocessing and analytic steps of the rs-fMRI scans*

All rs-fMRI images were processed in the FSL software (<https://fsl.fmrib.ox.ac.uk/fsl/fslwiki/FSL>) and Python (<https://www.python.org/>). The following preprocessing steps were applied: ① the first ten time points were removed; ② head motion correction: all volumes were realigned to the middle volume; ③ grand mean scaling; ④ spatial smoothing at a Gaussian kernel of 6mm full-width at half maximum; ⑤ the independent components analysis-based automatic removal of motion artifacts (ICA-AROMA) was applied to remove head motion artifacts further; ⑥ removing the cerebrospinal fluid and white matter signals by nuisance regression; ⑦ high pass filtering (0.01Hz); and ⑧ the preprocessed functional images were first registered to the high-resolution T1 images and finally to Montreal Neurological Institute (MNI152) standard space. Subjects with excessive head motion were excluded (>3mm of translation or >3 degrees of rotation in any direction).

**Results**

sTable 2: the demographic characteristics of the ADHD and the control group (sub-sample with fMRI images)

|  | ADHD | HC | t/F/χ^2^ statistics | *P* |
| --- | --- | --- | --- | --- |
| Sample size | 84 | 91 | N.A. | N.A. |
| Male | 68(80.95%) | 52((57.14%) | 11.49 | < 0.001 |
| Age | 8.76±1.28 | 9.31±1.31 | 2.80 | 0.0058 |
| Head motion | 0.067±0.025 | 0.070±0.039 | 0.54 | 0.59 |

Abbreviations: ADHD=attention deficit hyperactivity disorder; HC=healthy control.

sTable 3: reading skill and neuropsychological characteristics of the ADHD and the control group (sub-sample with fMRI images)

|  | measure | ADHD | HC | t/F/χ^2^ statistics | *P* |
| --- | --- | --- | --- | --- | --- |
| Chinese Reading Skill | CR | 87.32±30.84 | 104.40±21.50 | 7.60 | 0.0065 |
|  | OT | 26.48±7.25 | 31.03±5.99 | 11.08 | 0.0011 |
|  | WS | 12.40±6.68 | 17.31±6.17 | 13.68 | 0.00029 |
| WISC-IV | Full-scale IQ | 96.11±8.76 | 104.69±9.18 | 33.24 | <0.001 |
|  | VCI | 97.77±9.89 | 102.53±10.65 | 10.87 | 0.0012 |
|  | PRI | 102.80±8.62 | 106.47±10.41 | 4.64 | 0.033 |
|  | PSI | 95.62±9.95 | 107.36±11.57 | 39.29 | <0.001 |
|  | WMI | 90.69±9.48 | 97.29±9.99 | 17.42 | <0.001 |
| RVP | RVPA | 0.76±0.078 | 0.79±0.089 | 0.94 | 0.33 |
|  | RVPML | 631.35±161.23 | 583.97±109.63 | 3.85 | 0.052 |
| SWM | SWMS | 8.52±1.89 | 8.53±2.03 | 0.14 | 0.71 |
|  | SWMBE | 18.00±7.67 | 17.25±8.46 | 0.40 | 0.53 |
| SST | SSTSSRT | 386.95±69.27 | 342.82±82.16 | 6.71 | 0.011 |
|  | SSTDEG | 31.81±23.26 | 14.15±16.53 | 19.85 | < 0.001 |
|  | SSTDES | 51.45±8.66 | 47.15±8.08 | 6.08 | 0.015 |
| RTI | RTISMDRT | 413.89±66.47 | 382.91±63.64 | 3.54 | 0.062 |
|  | RTIFMDRT | 479.63±92.88 | 432.08±62.79 | 6.38 | 0.013 |
|  | RTISRTSD | 110.82±164.08 | 71.36±38.62 | 2.08 | 0.15 |
|  | RTIFRTSD | 100.47±56.26 | 87.40±51.85 | 0.35 | 0.56 |

Abbreviations: ADHD=attention deficit hyperactivity disorder; HC=healthy control; CR=Chinese character recognition; OT=orthographic knowledge (OT); WS=Word Chains; VCI=Verbal Comprehension Index; PRI=Perceptual Reasoning Index (PRI); WMI=Working Memory Index; PSI=Processing Speed Index (PSI); RVPA=Rapid Visual Information Processing (RVP), A prime; RVPML=Rapid Visual Information Processing (RVP), mean response latency; SWMS=Spatial Working Memory (SWM), strategy; SWMBE=Spatial Working Memory (SWM), between errors; SSTSSRT=Stop Signal Task (SST), stop signal reaction time; SSTDEG=Stop Signal Task (SST), direction errors: go trials; SSTDES=Stop Signal Task (SST), direction errors: stop trials; RTISMDRT=Reaction Time (RTI), reaction time of the simple mode; RTIFMDRT=Reaction Time (RTI), reaction time of the five-choice mode; RTISRTSD=Reaction Time (RTI), the standard deviation of reaction time of the simple mode; RTIFRTSD=Reaction Time (RTI), the standard deviation of reaction time of the five-choice mode;

sTable 4 Clusters showing between-group differences on brain functional connectivity

| Brain networks | comparisons | Cluster | Voxels | coordinates | regions |
| --- | --- | --- | --- | --- | --- |
| DMN | ADHD < HC | 1 | 68 | -22 4 14 | Left putamen; |
|  |  | 2 | 10 | -32 -20 6 | Left putamen; |
| ECN | ADHD < HC | 1 | 1445 | 56 -18 36 | Right precentral gyrus, right postcentral gyrus, right superior parietal lobule, right supramarginal gyrus, right lateral occipital cortex, posterior cingulate gyrus, precuneous cortex; |
|  |  | 2 | 358 | 32 -10 2 | Right putamen, right pallidum, right amygdala, right insular cortex, right planum polare; |
|  |  | 3 | 203 | 14 -14 0 | Right thalamus; |
|  |  | 4 | 188 | -30 -16 12 | Left putamen, left insular cortex; |
|  |  | 5 | 75 | 18 -10 20 | Right caudate, right thalamus; |
|  |  | 6 | 63 | 46 -4 44 | Right precentral gyrus; |
|  |  | 7 | 62 | 26 4 56 | Right superior frontal gyrus, right middle frontal gyrus; |
|  |  | 8 | 46 | 54 -2 12 | Right central opercular cortex; |
|  |  | 9 | 29 | 42 -12 54 | Right postcentral gyrus; |

Abbreviations: ADHD=attention deficit hyperactivity disorder; HC=healthy control; CR=Chinese character recognition; OT=orthographic knowledge (OT); DMN=default mode network; DAN=dorsal attention network;


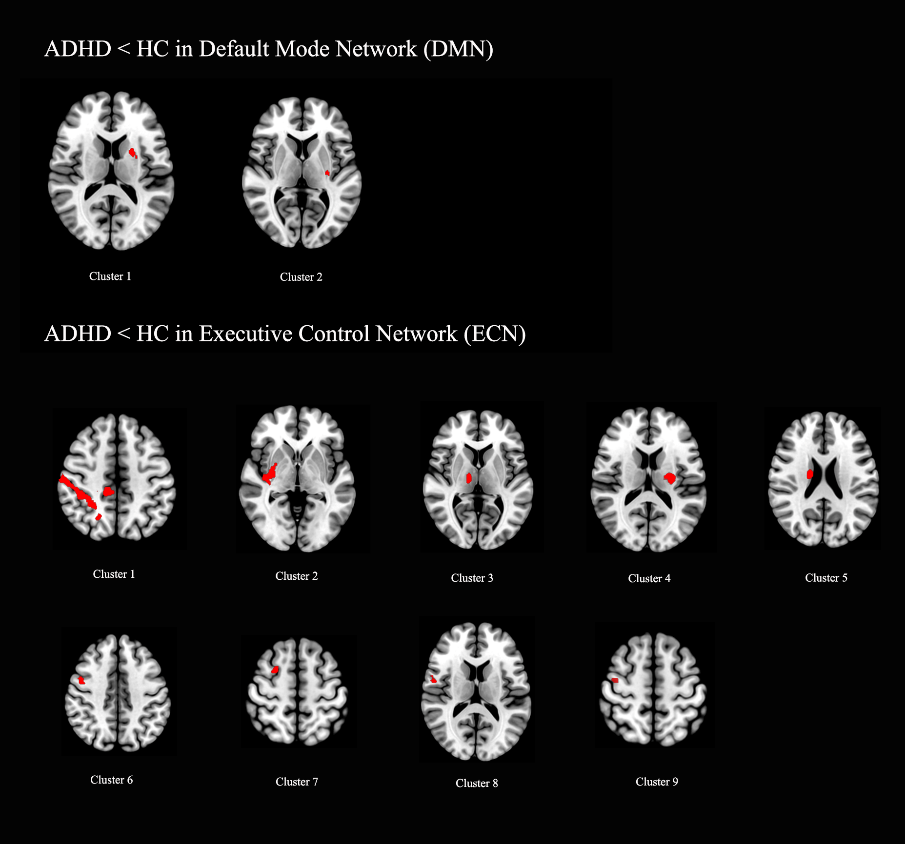


sFigure1: Comparisons of the strength of functional connectivity between subjects with ADHD and healthy controls, displayed on an MNI_T1_2mm_brain.

sTable 5 Clusters showing between-group differences on brain functional connectivity (with head motion as an additional covariate)

| Brain networks | comparisons | Cluster | Voxels | coordinates | regions |
| --- | --- | --- | --- | --- | --- |
| DMN | ADHD < HC | 1 | 14 | -22 4 14 | Left putamen; |
| ECN | ADHD < HC | 1 | 1083 | 56 -18 38 | Right precentral gyrus, right superior parietal lobule, right supramarginal gyrus, right lateral occipital cortex, precuneous cortex; |
|  |  | 2 | 899 | 30 -10 4 | Right thalamus, right putamen, right pallidum, right caudate, right amygdala, right insular cortex, right insular cortex, right planum polare; |
|  |  | 3 | 183 | -30 -16 12 | Left putamen, left insular cortex; |
|  |  | 4 | 40 | 14 -30 42 | Right precentral gyrus, right posterior cingulate gyrus |
|  |  | 5 | 21 | 36 2 8 | Right insular cortex; |

Abbreviations: ADHD=attention deficit hyperactivity disorder; HC=healthy control; CR=Chinese character recognition; OT=orthographic knowledge (OT); DMN=default mode network; DAN=dorsal attention network;

sTable 6 Clusters showing ADHD diagnosis-reading ability interacting effects (with head motion as an additional covariate)

| Brain networks | comparisons | Cluster | Voxels | coordinates | regions |
| --- | --- | --- | --- | --- | --- |
| DMN | CR_interaction | 1 | 2843 | 44 4 30 | Right frontal pole, right middle frontal cortex, right inferior frontal gyrus, right precentral gyrus, right central opercular cortex, right planum polare, right Heschl’s gyrus, right planum temporale; |
|  |  | 2 | 1678 | -42 18 16 | Left frontal pole, left middle frontal cortex, left inferior frontal gyrus, left precentral gyrus, left postcentral gyrus; |
|  |  | 3 | 687 | 42 -40 46 | Right postcentral gyrus, right superior parietal lobule, right supramarginal gyrus, right angular gyrus; |
|  |  | 4 | 348 | 34 -54 36 | right superior parietal lobule, right angular gyrus, right lateral occipital cortex; |
|  |  | 5 | 94 | 32 44 -2 | Right frontal pole; |
|  |  | 6 | 85 | -22 -6 50 | Left middle frontal cortex, left superior frontal gyrus, left precentral gyrus; |
|  |  | 7 | 79 | 34 -18 6 | Right insular cortex, right Heschl's gyrus |
|  |  | 8 | 72 | -12 4 46 | Left supplementary motor cortex, left anterior cingulate gyrus, left paracingulate gyrus; |
|  |  | 9 | 60 | 36 -14 -8 | Right insular cortex, right putamen; |
|  |  | 10 | 52 | 34 -18 64 | Right precentral gyrus; |
| DMN | OT_interaction | 1 | 1829 | -26 20 4 | Left frontal pole, left insular cortex, left middle frontal gyrus, left inferior frontal gyrus, left precentral gyrus, left frontal orbital cortex, left frontal operculum cortex, left central opercular cortex, left planum polare; |
|  |  | 2 | 351 | -2 14 24 | Anterior cingulate gyrus, left paracingulate gyrus; |
|  |  | 3 | 156 | 42 42 8 | Right frontal pole; right inferior frontal gyrus; |
|  |  | 4 | 14 | -12 6 -6 | Left putamen, left pallidum, left accumbens; |
| DAN | OT_interaction | 1 | 2216 | 46 -30 30 | Right postcentral gyrus, right superior parietal lobule, right supramarginal gyrus, right lateral occipital cortex, precuneous cortex, right parietal operculum cortex, right angular Gyrus; |
|  |  | 2 | 292 | -22 -52 52 | Left postcentral gyrus, left superior parietal lobule, left lateral occipital cortex; |
|  |  | 3 | 188 | -38 -48 36 | Left postcentral gyrus, left superior parietal lobule, left supramarginal gyrus, left angular gyrus; |
|  |  | 4 | 12 | 10 -74 10 | Right intracalcarine cortex; |
|  |  | 5 | 10 | 24 -24 48 | Right precentral gyrus; |

Abbreviations: ADHD=attention deficit hyperactivity disorder; HC=healthy control; CR=Chinese character recognition; OT=orthographic knowledge (OT); DMN=default mode network; DAN=dorsal attention network;
